# Numerical calculation of forward and reverse flow in Tesla valves with different longitudinal width-to-narrow ratios

**DOI:** 10.1038/s41598-023-39758-3

**Published:** 2023-08-01

**Authors:** Yu-Liang Zhang, Jiang-Bo Tong, Zu-Chao Zhu

**Affiliations:** 1 College of Mechanical Engineering and Key Laboratory of Air-Driven Equipment Technology of Zhejiang Province, Quzhou University, Quzhou, 324000 China; 2 School of Mechanical Engineering, Hunan University of Technology, Zhuzhou, 412007 China; 3 The Zhejiang Provincial Key Lab of Fluid Transmission Technology, Zhejiang Sci-Tech University, Hangzhou, 310018 China

**Keywords:** Energy science and technology, Engineering

## Abstract

To study the effect of the width-to-narrow ratio on the forward and reverse flow characteristics of the Tesla valve, five different models of the Tesla valve with different width-to-narrow ratios are established in this paper. The numerical calculations of forward and reverse flow under different working conditions are carried out by the CFD method in the laminar flow regime, and the reliability of the numerical calculation method is verified by comparing it with the experimental results. The results show that: in forward flow, the main flow-through channel is not related to the width-to-narrow ratio, the flow rate of the straight channel increases with the increase of the width-to-narrow ratio, and the static pressure in the diversion section is in the shape of “∞”; while in reverse flow, the main flow-through channel is weakly related to the width-to-narrow ratio, the flow rate of the arc channel is not increased with the increase of the width-to-narrow ratio, and the static pressure in the diversion section is in the shape of “bench”. As the width-to-narrow ratio decreases, the pressure drop during forward and reverse flow becomes more significant.

## Introduction

The Tesla valve is a passive check valve with no moving parts. Because it has no moving parts, there is less wear and tear while reducing the risk of clogging. In general, the resistance to flow in forward flow is less than in reverse flow, resulting in different flow characteristics in the forward and reverse flow and achieving the purpose of controlling fluid flow.

Many scholars have studied the prediction of flow characteristics and design applications of Tesla valves by numerical simulation or experiment. Regarding performance prediction, Topuz et al. conducted experiments on Al_2_O_3_, TiO_2_, and ZnO nanofluids flow in Tesla valve microchannels and investigated the effect of different pipes, temperatures, volume concentrations, and flow rates on the flow. The results show that Al_2_O_3_ nanofluid has the best thermal performance, and the heat transfer rate increases with the decrease of tube diameter^1^. Qian et al. studied the flow of Al_2_O_3_-water nanofluid in Tesla valves by numerical calculations. They discovered that the proportion of the mainstream increases with an increase in flow rate, temperature, and nanoparticle volume fraction^2^. Liosis et al. investigated the mixing efficiency of Fe_3_O_4_ nanoparticle fluid in the Tesla valve-type mixer and confirmed the inlet flow rate for better mixing by simulation^3^. Raffel et al. investigated the effect of Reynolds number on the flow in Tesla valve and visualized the velocity field using particle shadowgraph velocimetry (PSV). As the Reynolds number increases, the flow rate allocated in the main channel increases in the forward flow and decreases in the reverse flow; the diode of the Tesla valve increases with the flow rate^4^. Yontar et al. studied the flow characteristics of multi-stage Tesla valves under laminar and turbulent flow conditions. It is found that the pressures at inlet and outlet show different trends at different flow states^5^.

Chada et al. studied multi-stage Tesla valves at different angles and obtained maximum pressure, minimum pressure and maximum velocity at different Reynolds numbers. The CFD results show that the pressure required for reverse flow is higher than forward flow for the same flow rate^6^. Porwal et al. investigated the flow rectification and thermal enhancement capabilities of single and multi-stage Tesla valves by numerical calculations. They found that multi-stage Tesla valves have stronger heat transfer capability due to the characteristics of self-impingement, tortuous/mixing flow, stagnation, bifurcation, etc., and the thermal diode at high Reynolds numbers increases with the number of stages^7^. Mohammadzadeh et al. studied the effect of number of stages and stage diameter on the efficiency of Tesla valves. The research shows that the increase in the number of stages makes the fluid flow more complex but does not significantly improve the diode of the valve; the diode is independent of the valve passage diameter^8^. Hu et al. studied the Tesla valve at different angles and compared the diode properties of the Tesla valve at four different angles. At the same time, the flow field was analyzed by using the orthogonal decomposition method. It is found that the best diode performance of the Tesla valve is obtained in the 70°–80° and the diode characteristics of the Tesla valve are mainly generated by the separation bubble^9^. Liu et al. proposed a Tesla valve with a symmetric structure, and investigated the flow characteristics of symmetric and asymmetric Tesla valves at different Reynolds numbers. They discovered that the more symmetrical, the better the unidirectional flow characteristics of the Tesla valve system^10^.

For structural design and optimization, Vries et al. designed a new Tesla valve to promote pulsating heat pipe circulation and improve thermal resistance, and performed laminar single-phase model and steady two-phase flow experiments. It shows that the Tesla valve can promote pulsating heat pipe circulation^11^. Wang et al. proposed a new near zero-wear non-contact self-impact seal based on the passive fluid blocking principle and Tesla valve structure. It is found that the impact blocking effect of the three-dimensional leakage channel can achieve a stepwise throttling effect of the sealing medium, and increasing the seal stages and reducing the seal spacing can effectively control the leakage^12^. Cao et al. proposed a new fluid diode plate without moving parts to avoid contaminant backflow and reduce energy consumption, and performed both experimental and numerical simulations. This new plate has a much higher resistance to reverse flow than forward flow and can prevent or inhibit backflow^13^. Lai et al. designed a new interlayer battery thermal management system based on Tesla valve to improve the cooling efficiency of high power batteries, and investigated the effects of cold plate position and Tesla valve channel parameters on the cooling effect. They discovered that the thermal performance and cooling efficiency of the Tesla valve type channel are better than the linear type channel^14^. Lu et al. proposed a Tesla valve-type channel cold plate for rectangular lithium-ion batteries and analyzed the effects of the angle between adjacent Tesla valves, the distance between adjacent Tesla valves, the distance between adjacent channels, and the coolant inlet velocity on the battery by numerical simulations. It is found that the reverse Tesla valved channel cold plate with angle of 120°, Tesla valve distance of 23.1 mm, channel distance of 28 mm, and inlet velocity of 0.83 m/s possesses a good balance between heat transfer performance and energy consumption^15^. Wang et al. devised a Tesla valve type micromixer and found that this new micromixer has low pressure drop and high mixing performance during the mixing process. Meanwhile, they obtained the optimal geometric parameters of the Tesla-type micro-mixer by studying^16^.

In summary, Tesla valves have been studied in-depth in terms of fluid types and nature, geometric parameterization effects, and structural design applications. However, the difference in flow performance of the Tesla valve caused by the variation of the flow channel width-to-narrow ratio has not been thoroughly and systematically studied. Based on this, Tesla valve models with five different width-to-narrow ratios are established in this paper. Numerical calculations of forward and reverse flows are carried out in the laminar flow regime. By calculation, the flow characteristics at different width-to-narrow ratios are obtained, and the performance effects caused by changes in the width-to-narrow ratio of the flow channel are grasped. This paper aims to investigate the effect of width-to-narrow ratio on the performance of Tesla valve so as to optimize the geometry of Tesla valve and obtain high performance, which provides some reference significance for the application of Tesla valve in flow control.

## Physical models and computational method

### Calculation models

**Figure 1:**
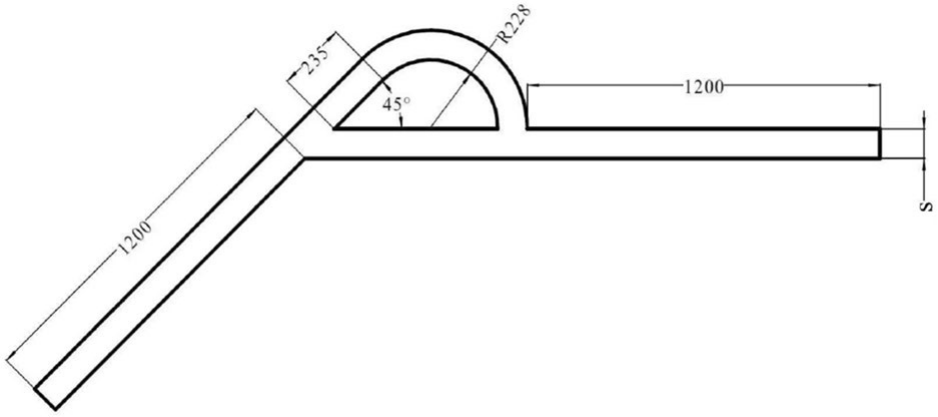
Tesla model.

**Figure 2:**
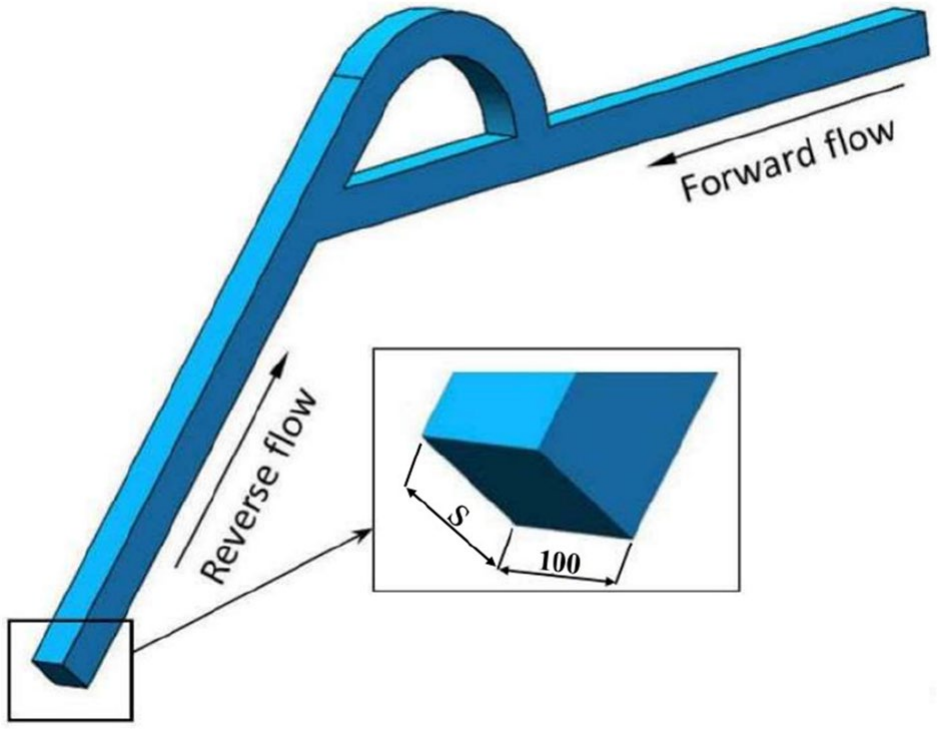
Three-dimensional model.

**Figure 3:**
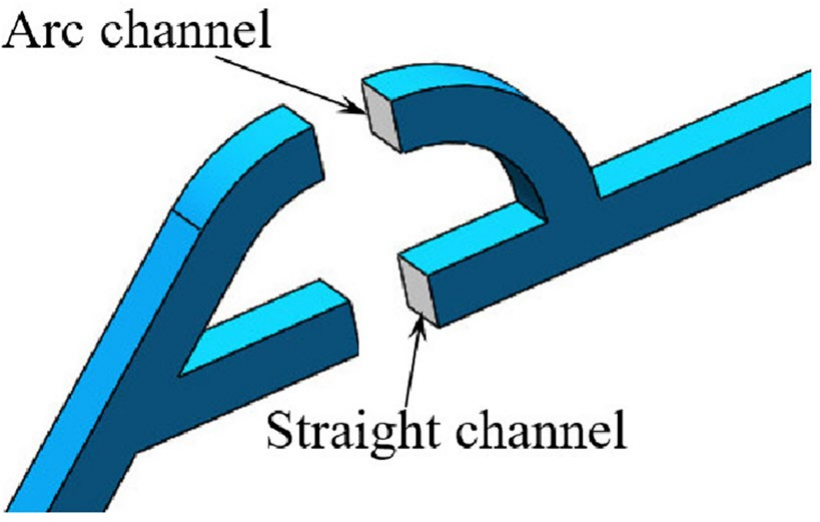
Arc and straight channels.

The structure of the Tesla valve in this paper is shown in Fig. 1 in µm. By changing the width *s*, Tesla valves with different width-to-narrow ratios are obtained. When the fluid flows into the valve from the straight channel, the resistance is small, it is the forward flow. When the fluid flows into the valve from the inclined channel, the resistance is larger, it is the reverse flow. The Tesla valve flow definition and 3D model are shown in Fig. 2, and the arc channel and straight channel are shown in Fig. 3. The width-to-narrow ratio is defined as in Eq. (1), where b = 100 μm.

1 $$ \alpha = \frac{s}{{\text{b}}} $$

### Calculation scheme

**Figure 4:**
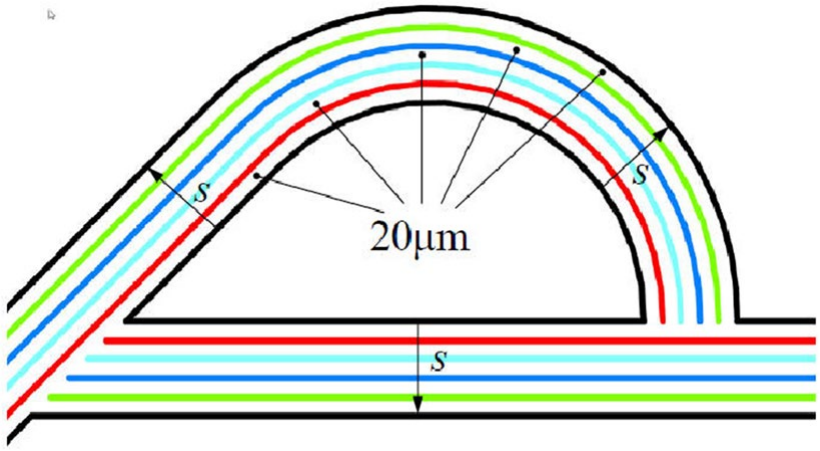
Evolution schematic.

**Figure 5:**
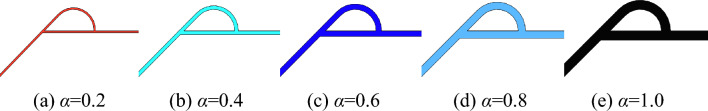
Calculation domains of Tesla valves for different width-to-narrow ratio scenarios.

In this paper, the inner wall of the diversion section of the five Tesla valve models is the same size. Based on the inner wall, the width *s* is gradually increased from inside to outside to obtain Tesla valves with different width-to-narrow ratios. Figure 4 shows the variations of the model. Accordingly, five kinds of Tesla valves with different width-to-narrow ratios are obtained, and the calculation domains are shown in Fig. 5.

By simulating the forward and reverse flows at inlet flow rate from 1 mL/min to 8 mL/min, the through-flow capacity of Tesla valve with different width to narrow ratio is obtained. At the same time, numerical simulations of forward and reverse flows at incoming velocities of 5 m/s, 10 m/s, 15 m/s and 20 m/s are also carried out in this paper, and the characteristics of pressure drop characteristics, velocity variation and vortex distribution are finally obtained.

### Calculation method

As a non-circular flow section, its hydraulic diameter is

2 $$ d_{{\text{e}}} = 4\frac{A}{X} $$

where* A* is the overflow cross-sectional area, and *X* is the wet perimeter.

Based on Reynolds number calculation

3 $$ Re = \frac{{vd_{{\text{e}}} }}{\gamma } $$

where *v* is the inlet velocity, $\gamma$ is the kinematic viscosity, the flow medium in this paper is water, taking its kinematic viscosity as 1 × 10^-6^m^2^/s.

The results of the Reynolds number calculations for each calculation model in this paper at the maximum calculated flow rate (8 mL/min) and the maximum calculated velocity (20 m/s) are shown in Table 1. In which, Re(max_1_) means the Reynolds number of the model under the maximum calculated flow rate for each width-to-narrow ratio case, and Re(max_2_) means the Reynolds number of the model under the maximum calculated velocity for each width-to-narrow ratio case.

**Table 1:** Reynolds number.

No	S (μm)	*α*	Re (max_1_)	Re (max_2_)
1	20	0.2	2221.67	666.67
2	40	0.4	1904.29	1142.85
3	60	0.6	1666.25	1500
4	80	0.8	1480.89	1777.78
5	100	1.0	1333	2000

As can be seen, the maximum Reynolds number is 2221.67 under five width-to-narrow ratio cases, and the fluid flow state under all calculation schemes is laminar. In this paper, ANSYS-FLUENT is used to calculate the steady, incompressible and three-dimensional viscous flow in the Tesla valve. The governing equation to be solved is

4 $$ \partial_{{u_{j} }} /\partial_{{x_{j} }} = 0 $$

5 $$ u_{j} \frac{{\partial u_{i} }}{{\partial x_{j} }} = f_{i} - \frac{1}{\rho }\frac{{\partial_{p} }}{{\partial x_{i} }} + \frac{\mu }{\rho }\frac{\partial }{{\partial x_{j} }}\left( {\frac{{\partial u_{i} }}{{\partial x_{j} }} + \frac{{\partial u_{j} }}{{\partial x_{i} }}} \right) $$

**Figure 6:**
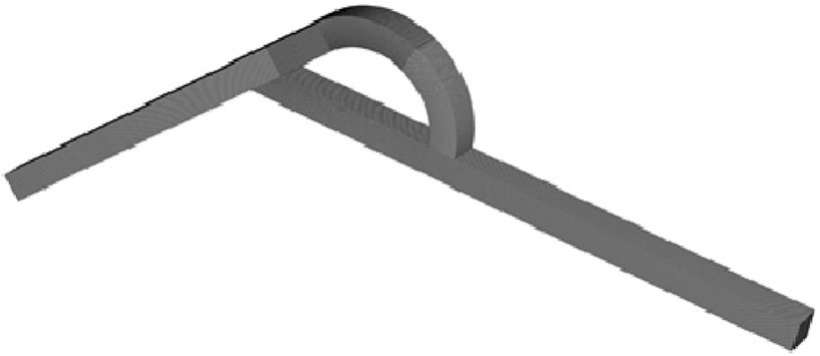
The computational meshes of the Tesla valve at α = 1.0.

Based on ICEM, the hexahedral mesh is used to divide each model structure. Qian et al. have studied this type Tesla valve numerically and performed mesh independence checks. The relative error of diode is 0.88% when the total mesh number is 967,353 and 1,043,610, indicating that the 967,353 meshes are sufficient for accurate simulation^2^. Therefore, the calculated mesh number of the Tesla valve model is 194,688, 388,746, 595,850, 802,467 and 1,042,470 under the five width-to-narrow-ratio conditions (*α* = 0.2, 0.4, 0.6, 0.8 and 1.0), which is sufficient. The computational meshes of the Tesla valve at α = 1.0 are shown in Fig. 6. The flowing medium in this calculation is liquid water, which is solved by a three-dimensional double-precision solver. The velocity-inlet boundary is adopted at the forward and reverse inlet of the Tesla valve, which is determined according to the flow rate and the cross-section area. The pressure-out boundary is adopted at the outlet.

## Analysis of results

### Calculation validation

In this paper, four kinds of calculation models and corresponding calculation cases in the literature^1^ are used for calculation validation. The calculation model and scheme are shown in Table 2, and the comparison between the calculation results and experimental results is shown in Table 3. The theoretical calculation results in Table 3 are calculated using the Darcy-Weisbach formula. It can be found that the maximum relative error between the calculated results and the experimental values is -8.11% and the minimum error is 3.33%. Therefore, the numerical calculation method adopted in this paper is considered reliable, and the calculated results are acceptable. The numerical calculation of internal flow in the Tesla valve will be carried out based on this method. It is also found that the theoretical values calculated based on the Darcy-Weisbach formula are smaller than the experimental values.

**Table 2:** Calculation models.

No.	Tube inner diameter *d*(μm)	Tube length *l*(cm)	Fluid media	Volume concentration (%)	Flow rate (mL/min)
1	400	20	Al_2_O_3_ nanofluid	1.0	35
2	750	20	ZnO nanofluid	1.0	35
3	1000	20	Al_2_O_3_ nanofluid	1.0	20
4	1000	20	ZnO nanofluid	0.5	20

**Table 3:** Comparison of pressure drops.

No.	Experiment (MPa)	Numerical calculation (MPa)	Theory (MPa)	Errors between Numerical calculations and experiments (%)
1	0.2070	0.1992	0.1857	− 3.77
2	0.0167	0.0179	0.0150	7.19
3	0.0030	0.0031	0.0027	3.33
4	0.0037	0.0034	0.0027	− 8.11

### Flow-through capacity

**Figure 7:**
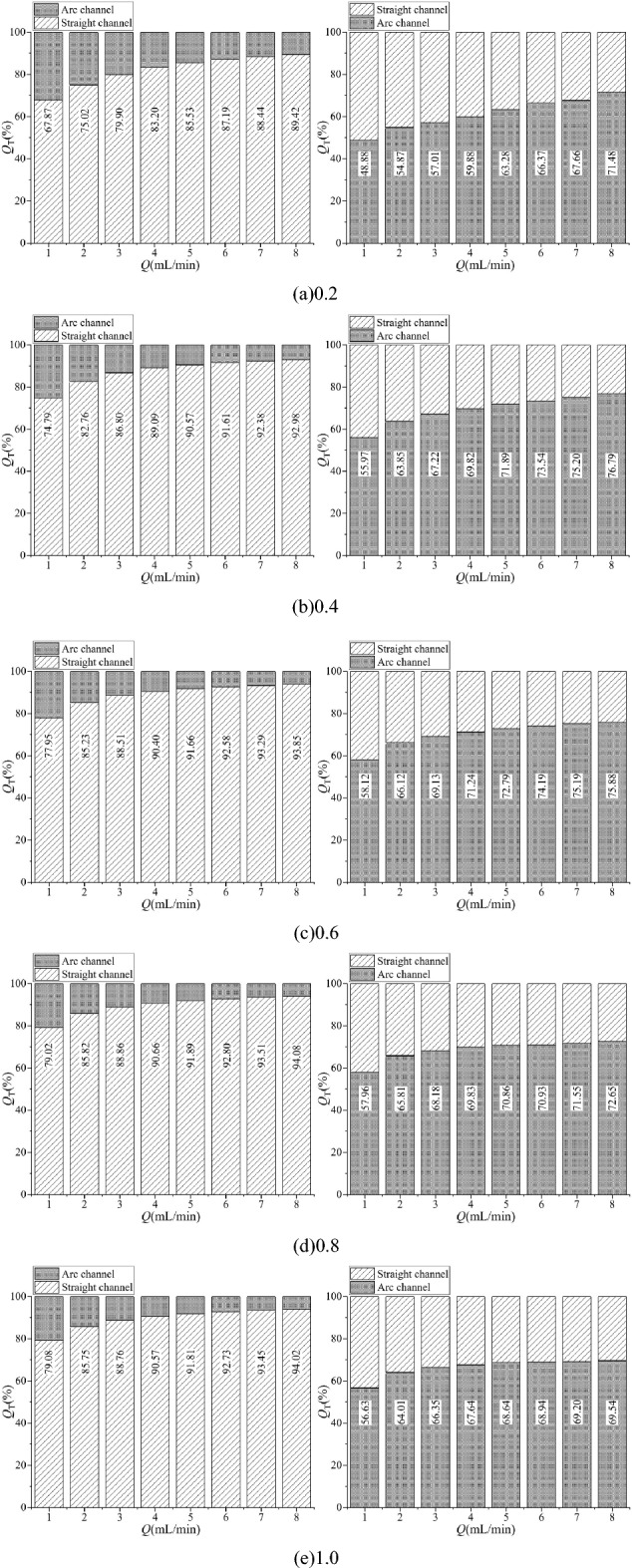
Proportion of flow distribution for different width-to-narrow ratio scenarios (left: forward flow; right: reverse flow).

The multi-channel design of the Tesla valve allows different flow distribution in each channel inside it, and the fluids in each channel interact with each other to achieve flow control. In different situations, the flow allocation of the arc and straight channels are not the same. The flow allocation is shown in Fig. 7, where *Q*_T_ denotes the proportion of the total flow rate allocated to the arc and straight channels.

In forward flow, most of the fluid in the valve flows through the straight channel, and the straight channel is the main channel. For example, in the case of width-to-narrow ratio *α* = 0.2, when the total flow rate is 1, 2, 3, 4, 5, 6, 7, 8 mL/min, the corresponding flow proportion through the straight channel is 67.87%, 75.02%, 79.90%, 83.20%, 85.53%, 87.19%, 88.44%, 89.42%, while the corresponding flow proportion through the arc channel is 32.13%, 24.98%, 20.10%, 16.80%, 14.47%, 12.81%, 11.56%, 10.58%, respectively. As can be seen, the flow proportion through the straight channel is much higher than that through the arc channel. For all other width-to-narrow ratio cases, the flow rate through the straight channel at any flow rate is also higher than that of the arc channel. In conclusion, the main flow-through channel in the forward flow is always the straight channel.

Unlike forward flow, in reverse flow, most of the fluid flows through the arc channel in most cases. However, at small flow rates and low width-to-narrow ratio, most fluid flows through the straight channel, and the main flow-through channel is not always an arc channel. In the width-to-narrow ratio *α* = 0.2, when the total passing flow rate is 1 mL/min, the corresponding flow proportion through the arc channel is 48.88%, while the flow proportion through the straight channel reaches 51.12%, the flow rate through the latter is higher than that of the former. Obviously, the main flow-through channel is the straight channel in this condition. Moreover, at the same total through flow rate of 1 mL/min, when *α* = 0.4, 0.6, 0.8, 1.0, the corresponding flow proportion through the arc channel is 55.97%, 58.12%, 57.96%, 56.63%, the main flow-through through channels are arc channel. As can be seen, in the reverse flow of small flow rate and low width-to-narrow ratio, the flow-through capacity of the straight channel is higher than that of the arc channel; while in other cases, the arc channel is still the main flow-through channel.

In addition, it is also found that the flow-through capacity of the straight channel as the main flow-through channel during forward flow is much greater than that of the arc channel as the main flow-through channel during reverse flow. For example, in the case of width-to-narrow ratio *α* = 0.8, when the total flow rate is 1, 2, 3, 4, 5, 6, 7, 8 mL/min, the corresponding flow proportion through the straight channel in forward flow is 79.02%, 85.82%, 88.86%, 90.66%, 91.89%, 92.80%, 93.51%, 94.08%; the corresponding flow proportion through the arc channel in reverse flow is 57.96%, 65.81%, 68.18%, 69.83%, 70.86%, 70.93%, 71.55%, 72.65%, respectively. Obviously, by the special structure of the Tesla valve, the flow loss in the reverse flow is greater than that in the forward flow, which leads to the main flow-through channel in the reverse flow being weaker than the main flow-through channel in the forward flow.

In forward flow, as the flow rate increases, the flow proportion through the straight channel gradually increases, and the flow proportion through the arc channel gradually decreases. For example, in the case of width-to-narrow ratio *α* = 0.4, when the total through flow rate changes from 1 to 8 mL/min, the flow proportion through the straight channel increases from 74.79 to 92.98%, while the corresponding flow proportion through the arc channel decreases from 25.21 to 7.02%. While the reverse flow, with the increase in the flow rate, the flow proportion through the arc channel gradually increases, and the flow proportion through the straight channel gradually decreases. In the case of width-narrow ratio *α* = 0.4, when the total through flow rate changes from 1 to 8 mL/min, the flow proportion through the straight channel increases from 55.97 to 76.79%, while the corresponding flow proportion through the arc channel decreases from 44.03 to 23.21%. As the total flow rate increases, the flow allocation within the two channels in the forward and reverse flow shows an opposite trend.

For forward flow, in the small flow rate range, the flow rate occupied by the straight channel increases significantly as the flow rate increases. In contrast, the flow rate occupied by the straight channel does not increase significantly in the big flow rate range. For example, at *α* = 0.6 for forward flow, when the total through flow rate increases from 1 to 4 mL/min, the corresponding flow proportion through the straight channel increases from 77.95 to 90.40%, with a difference of 12.45%. Furthermore, when the flow rate increases from 5 to 8 mL/min, the flow proportion through the straight channel increases from 91.66 to 93.85%, with a difference of only 2.19%. In the reverse flow, as the flow rate increases, the proportion of the flow rate occupied by the arc channel increases significantly in the small flow rate range; and the proportion of the flow rate occupied by the arc channel does not increase much in the big flow rate range. In summary, at small flow rates, the flow rate allocated in the main channel changes more rapidly and is sensitive to the response to flow rate changes. In contrast, when the flow rate is high, the flow rate allocated in the main channel changes more slowly and is not sensitive to the response to flow rate changes.

For the same flow rate in the forward flow, the proportion of flow rate occupied by the straight channel tends to increase as the width-to-narrow ratio increases. In all the forward calculation schemes in this paper, except for the total flow rate of 2 mL/min, the flow rate proportion of the straight channel at all flow rates increases gradually with the increase of the width-to-narrow ratio. At the flow rate of 2 mL/min, the corresponding proportion of the flow rate occupied by the straight channel at *α* = 0.2, 0.4, 0.6, 0.8, and 1.0 is 75.02%, 82.76%, 85.23%, 85.82%, and 85.75%, respectively. At this flow rate, the proportion of flow rate in the straight channel does not increase gradually with the increase of the width-to-narrow ratio, but the overall trend shows an increase.

In the forward flow, the variation of the width-to-narrow ratio in the low width-to-narrow ratio range significantly affects the flow at the same total flow rate. When the total through flow rate is 1, 2, 3, 4, 5, 6, 7, and 8 mL/min, the corresponding flow proportion through the straight channel at *α* = 0.2 is 67.87%, 75.02%, 79.90%, 83.20%, 85.53%, 87.19%, 88.44%, and 89.42%, respectively, and the corresponding flow proportion through the straight channel at *α* = 0.4 is 74.79%, 82.76%, 86.80%, 89.09%, 90.57%, 91.61%, 92.38%, and 92.98%, respectively. It can be seen that at each flow rate, as the width-to-narrow ratio *α* = 0.2 increases to *α* = 0.4, the flow proportion through the straight channel increases, and the increase is more evident in the low flow rate range. However, the flow allocation of the Tesla valve for different width-to-narrow ratio cases at the same flow rate in the width-to-narrow ratio *α* = 0.6 to *α* = 1.0 are not significantly different. To sum up, the flow of the Tesla valve in the low width-narrow ratio range responds sensitively to the variation of the width-to-narrow ratio.

For reverse flow, the change in the flow channel width-to-narrow ratio has a different effect on the flow and allocation of fluid in the valve than when flowing in the forward direction. The increase in the width-to-narrow ratio does not result in a trend of increasing flow proportion through the main flow-through channel. At the flow rate of 3 mL/min, the corresponding flow proportion through the arc channel at *α* = 0.2, 0.4, 0.6, 0.8, and 1.0 is 57.01%, 67.22%, 69.13%, 68.18%, and 66.35%, respectively. At the flow rate of 8 mL/min, the corresponding flow proportion through the arc channel at *α* = 0.2, 0.4, 0.6, 0.8, and 1.0 is 71.48%, 76.79%, 75.88%, 72.65%, and 69.54%, respectively. It can be seen that, with the increase of the width-to-narrow ratio of the flow channel, the proportion of the flow rate in the arc channel shows a trend of increasing and then decreasing, and the maximum value appears mainly in the case of *α* = 0.4, 0.6.

Similar to the forward flow, the flow of the Tesla valve is also sensitive to changes in the width-to-narrow ratio in the low width-to-narrow ratio range when flowing in the reverse direction. When the total flow rate is 1, 2, 3, 4, 5, 6, 7, and 8 mL/min, the corresponding proportion of arc channel flow rate at *α* = 0.2 is 48.88%, 54.87%, 57.01%, 59.88%, 63.28%, 66.37%, 67.66%, and 71.48%, and the corresponding proportion of arc channel flow rate at *α* = 0.4 is 55.97%, 63.85%, 67.22%, 69.82%, 71.89%, 73.54%, 75.20%, and 76.79%. The proportion of the flow rate occupied by the arc channel changes significantly as the width-to-narrow ratio increases in this width-to-narrow ratio range.

### Pressure drop characteristics

**Figure 8:**
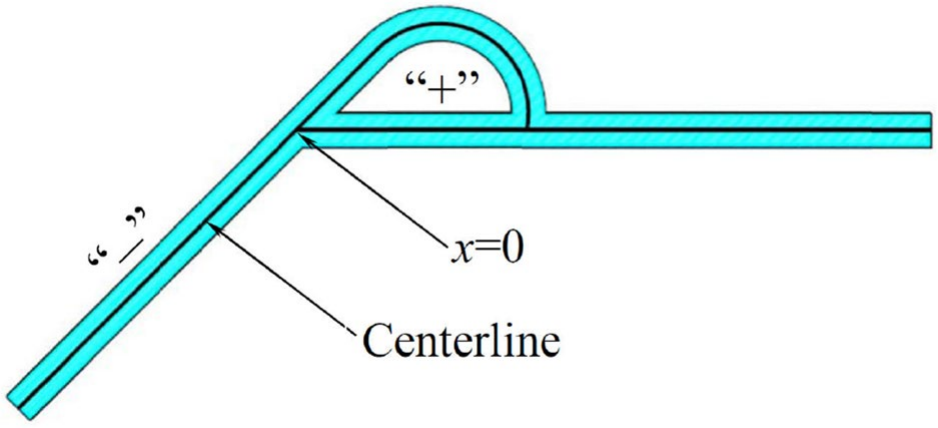
Pressure monitoring centreline.

**Figure 9:**
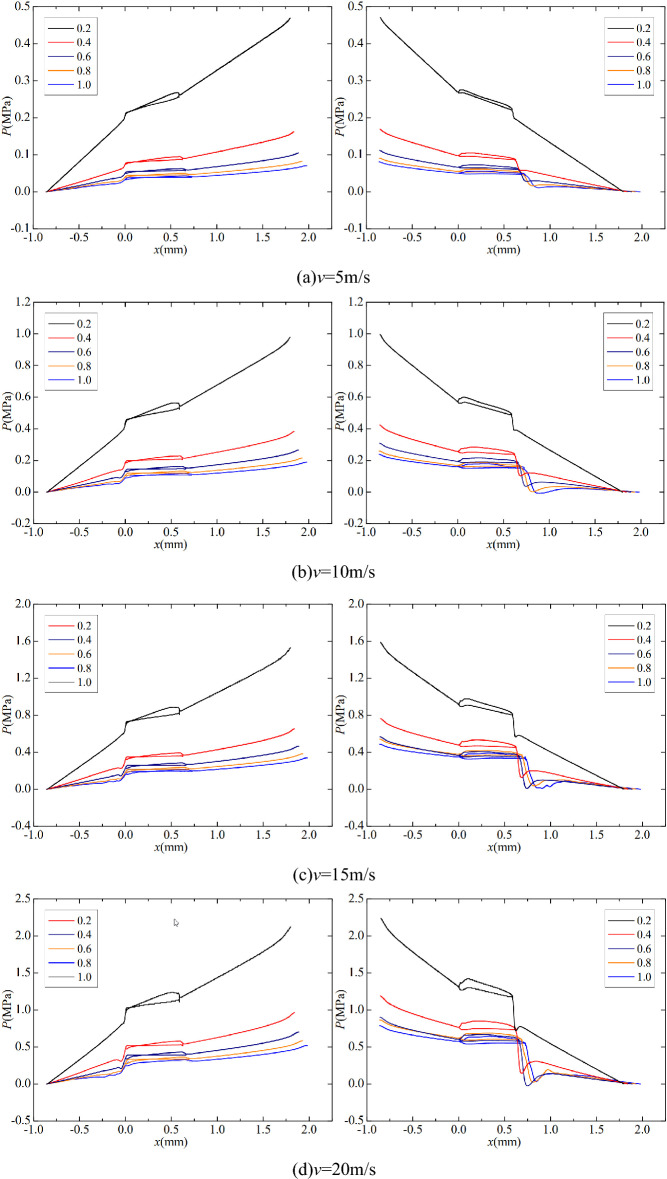
Centreline pressure for different incoming flow velocitys (left: forward flow; right: reverse flow).

The pressure drop is an important measure of the flow performance of the Tesla valve. In this paper, the forward and reverse flows at incoming velocities of 5 m/s, 10 m/s, 15 m/s and 20 m/s are numerically simulated to obtain the static pressure variation curves at the centerline of the Tesla valve. Figure 8 shows the monitoring centerline established in the simulation. The straight channel centerline intersects with the inclined channel centerline at “x = 0”, the left inclined channel centerline is “ − ”, and the right straight channel and the arc channel centerlines are both “ + ”. Figure 9 shows the static pressure distribution characteristics of the centerline in the Tesla valves at different incoming flows.

Regardless of the forward or reverse flow, there is an overall decreasing pressure trend from the valve inlet to the outlet. For the case of *v* = 5 m/s and *α* = 0.2, the pressure drops from 0.469 to 0 MPa for forward flow and drops from 0.471 to 0 MPa for reverse flow. As the fluid flows through the valve, the flow loss increases, and the pressure decreases from the inlet to the outlet.

At the same time, it is found that the pressure in the valve does not decrease gradually with the flow and does not show a monotonic decreasing trend. For example, in the case of forward flow at *v* = 10 m/s, when *α* = 0.2, the static pressure in the channel first drops from 0.979 to 0.544 MPa, then rises to 0.564 MPa in the straight channel and finally drops to 0 MPa at the outlet; While the reverse flow under the same condition, the static pressure in the channel first drops from 0.997 to 0.573 MPa, then rises to 0.599 MPa in the arc channel, then drops to 0.390 MPa at the exit of the diversion section, then rises to 0.392 MPa, and finally drops to 0 MPa at the exit. As can be seen, due to the part of the kinetic energy turns into pressure energy, there is pressure rebound in some areas during both forward and reverse directions.

In addition, under the same conditions, the valve inlet pressure is greater for reverse flow than forward flow. For example, at *v* = 5 m/s, for five width-to-weight ratio cases (*α* = 0.2, 0.4, 0.6, 0.8, 1.0), the corresponding inlet pressures for forward flow is 0.469 MPa, 0.162 MPa, 0.105 MPa, 0.083 MPa, and 0.071 MPa, the corresponding inlet pressures for reverse flow is 0.471 MPa, 0.169 MPa, 0.112 MPa, 0.091 MPa, and 0.081 MPa, while the pressures at their outlets are all 0 MPa. It is clear that the pressure drop is slightly greater in the reverse flow than in the forward flow for each width-to-narrow ratio. Also, it is found that as the incoming velocity increases, the pressure drop difference between the forward and reverse flow is greater. When the incoming flow velocity increases to *v* = 20 m/s, the corresponding inlet pressures is 2.120 MPa, 0.964 MPa, 0.702 MPa, 0.585 MPa, 0.521 MPa for forward flow and 2.240 MPa, 1.190 MPa, 0.902 MPa, 0.866 MPa, 0.788 MPa for reverse flow in the five width-narrow ratio cases (*α* = 0.2, 0.4, 0.6, 0.8, and 1.0). Evidently, the pressure drop is greater at high incoming velocities, and the difference between forward and reverse flow is greater.

At the same inlet velocity, the static pressure in the valve is enhanced overall as the flow channel width-to-narrow ratio decreases. For example, at *v* = 15 m/s, in five width-narrow ratio cases (*α* = 0.2, 0.4, 0.6, 0.8, 1.0), the corresponding inlet pressures is 1.530 MPa, 0.654 MPa, 0.467 MPa, 0.384 MPa, 0.339 MPa for forward flow and 1.590 MPa, 0.764 MPa, 0.566 MPa, 0.541 MPa, 0.489 MPa for reverse flow. Obviously, the inlet pressure at a low width-to-narrow ratio is greater than that at a high width-to-narrow ratio. It is also found that the inlet pressure increases significantly with decreasing width-to-narrow ratio in the low width-to-narrow ratio range. When *α* = 0.4 is reduced to *α* = 0.2, the inlet pressure increases by 0.876 MPa for forward flow and 0.826 MPa for reverse flow. However, when *α* = 1.0 is reduced to *α* = 0.8, the inlet pressure increases by only 0.045 MPa for forward flow and 0.052 MPa for reverse flow.

In the Tesla valve, the most critical part is the diversion section, i.e. the straight channel and the arc channel in the middle of the Tesla valve. It is the diversion section that allows the Tesla valve to have different flow characteristics in the forward and reverse flow for flow control. Compared with the other parts, the flow in and around the diversion section is more complex. Thus, the diversion section's pressure distribution characteristics differ dramatically during forward and reverse flow.

For forward flow, the static pressure varies in a “∞” shape in the diversion section. In the case of *v* = 10 m/s, with *α* = 0.4, the static pressure in the straight channel slowly rises from 0.218 to 0.229 MPa and then slowly decreases to 0.185 MPa; while the static pressure in the arc channel plummets from 0.218 to 0.205 MPa, then slowly decreases to 0.197 MPa and finally plummets to 0.185 MPa. In other cases of forward flow calculations, the pressure in the diversion section also varies in the shape of “∞”, and the area to the right of “∞” is significantly larger than the area to the left. It can be seen that at the equal “x”, in the front diversion section, the static pressure in the straight channel is higher than that in the arc channel; in the latter diversion section, the static pressure in the arc channel is higher that in than the straight channel.

In addition, the right area of “∞” is larger than the left area, indicating that the pressure difference between the straight channel and the arc channel in the front diversion section is significantly larger than the pressure difference in the latter diversion section. The pressure loss is mainly in the front diversion section. Under the same conditions, as the width-to-narrow ratio decreases, the right side of “∞” is significantly larger, and the left side is even close to overlapping. As seen in all calculated cases of forward flow in this paper, the difference of static pressure in the front diversion section is greater for the low width-to-narrow ratio. As the width-to-narrow ratio decreases, more flow energy is lost in the front diverging section.

For forward flow, the pressure drop is greater at a low width-to-narrow ratio after flowing through the diversion section. For example, at* v* = 20 m/s, for five width-to-narrow ratio cases (*α* = 0.2, 0.4, 0.6, 0.8, 1.0), the corresponding pressure at the start diversion point is 1.190 MPa, 0.551 MPa, 0.410 MPa, 0.347 MPa, 0.314 MPa, the corresponding pressure at the end diversion point is 0.963 MPa, 0.481 MPa, 0.348 MPa, 0.283 MPa, 0.248 MPa, and the pressure drop is 0.227 MPa, 0.070 MPa, 0.062 MPa, 0.064 MPa, 0.066 MPa, respectively. The pressure drop at *α* = 0.2 is much higher than at other width-narrow ratios. Not only in the diversion section but also in the whole flow region, the pressure drop at *α* = 0.2 is the most significant. Therefore, the width-to-narrow ratio of the Tesla valve should be as small as possible for large pressure drop requirements.

However, for reverse flow, the static pressure varies in a “bench” shape in the diversion section, which differs from the forward flow. In other words, at equal “x”, the static pressure in the arc channel is always higher than the static pressure in the straight channel. As the reverse flow at the incoming velocity of 10 m/s, under the case of the width-to-narrow ratio *α* = 0.4, the static pressure in the arc channel first rises from 0.257 to 0.280 MPa and then falls to 0.232 MPa; In contrast, the static pressure in the straight channel decreases monotonically from 0.257 to 0.232 MPa. In addition, for reverse flow, the pressure drop after the fluid flows through the diversion section is greater as the width-to-narrow ratio decreases. Like the forward flow, the pressure drop is also most significant at *α* = 0.2.

Meanwhile, it is found that regardless of the forward and reverse flow, there is a pressure plunge after flowing through the diversion section, and the plunge is more significant in the reverse flow. Such as forward flow at 10 m/s, in the case of width-to-narrow *α* = 0.2, the static pressure of the Tesla valve at the end diversion point is about 0.435 MPa, while the static pressure at the end plunge point in the inclined channel is about 0.402 MPa, the static pressure curve is close to the vertical drop. Under the same conditions, the static pressure at the end diversion point is about 0.481 MPa for reverse flow, while the static pressure at the end plunge point in the inclined channel is about 0.391 MPa. Clearly, the static pressure curve at reverse flow also falls nearly vertically, and the plunge is greater.

### Velocity distribution

**Figure 10:**
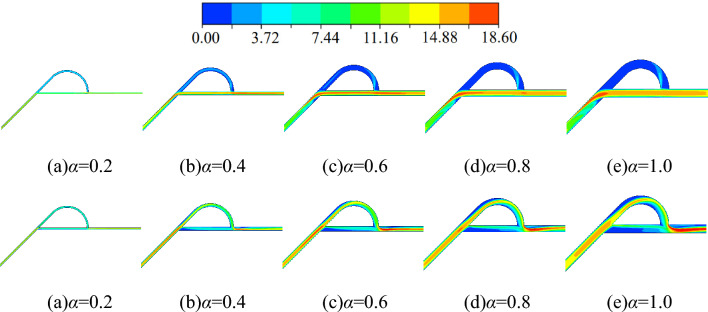
Velocity clouds for different width-to-narrow ratio scenarios (m/s) (above: forward flow; below: reverse flow).

No matter the forward or reverse flow, the fluid flowing into the Tesla valve is divided and then combined by the action of the diverter section. The fluid in the arc channel interacts with the fluid in the straight channel, and the internal flow is complex. Therefore, it is necessary to analyze its internal flow velocity. Figure 10 shows the forward and reverse flow velocity clouds for different width-to-narrow ratio cases, and the incoming flow velocity is 10 m/s.

In the five width-to-narrow ratio cases (*α* = 0.2, 0.4, 0.6, 0.8, 1.0), for forward flow, the maximum flow velocities in the middle straight channel of the Tesla valve is about 13.02 m/s, 16.74 m/s, 16.74 m/s, 16.74 m/s and 16.74 m/s, respectively. In contrast, the maximum flow velocities in the middle arc channel is about 5.58 m/s, 3.72 m/s, 1.86 m/s, 1.86 m/s and 1.86 m/s, respectively. As can be seen, the maximum flow velocity in the middle straight channel is much larger than that in the middle arc channel at each width-to-narrow ratio. For the reverse flow, the velocity cloud in Fig. 10 shows that the flow velocity in the arc channel is much larger than that in the straight channel, i.e., the flow rate in the arc channel is much larger than that in the straight channel. It is seen that the velocity cloud graphs in Fig. 10 and the flow allocation graphs in Fig. 7 show the same flow conditions and verify each other.

In forward flow, a small part of the fluid enters the arc channel and impacts the inner wall of the diversion section, forming the jet in the arc channel. In the low width-to-narrow ratio cases, the jet intensity is higher, and the jet affects the whole arc channel. As seen when *α* = 0.2, the maximum jet velocity is about 14.88 m/s, and the jet is distributed in the whole arc diversion section. As the width-to-narrow ratio increases, the jet intensity and the area of jet influence in the arc channel decrease significantly. As seen when *α* = 1.0, the maximum velocity of the jet is about 5.58 m/s, and the jet is only distributed in the front section of the arc diversion channel.

What's more, most of the fluid flows into the straight channel in the forward flow, and due to the sudden transition from the straight channel to the inclined channel, a large amount of fluid impacts the valve wall, creating a jet at the exit of the diversion section and the inclined channel. As seen at *α* = 0.2, the maximum jet flow velocity is about 16.74 m/s, and the jet is distributed throughout the inclined channel. While *α* = 1.0, the maximum jet flow velocity is about 18.60 m/s, and the jet is only distributed at the outlet of the diversion section. Obviously, the jet intensity increases as the width-to-narrow ratio increases, but the area of jet influence reduces.

For reverse flow, the jet also exists at the exit of the diversion section. The large amount of fluid in the arc channel and the sudden flow transition from the arc channel to the straight channel also produce a jet in the straight channel at the exit of the diversion section. As seen in reverse flow, the maximum velocity of the jet increases as the width-to-narrow ratio increases, while the jet area is always the entire straight channel. It indicates that the jet intensity in this region increases with the increase of the width-to-narrow ratio, and the jet region is unresponsive to the width-to-narrow ratio.

### Vorticity characteristics

**Figure 11:**
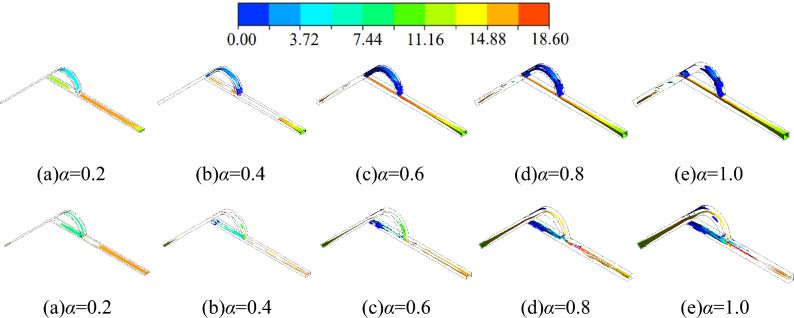
Vorticity clouds for different width-to-narrow ratio scenarios (above: forward flow; below: reverse flow).

Figure 11 shows the vorticity cloud at an incoming velocity of 10 m/s, where the vortex intensity is 64,670.8 s^-1^. In forward flow, the vorticity in the low width-to-narrow ratio is distributed throughout the whole flow channel. It is mainly distributed in the straight channel and the diversion section, with only a tiny part in the inclined channel. With the increase of the width-to-narrow ratio, the vorticity at the exit of the arc diversion section is gradually reduced, and even the vorticity of this region disappears completely when *α* = 1.0. However, the variation of the vorticity in the inclined channel shows a different trend. With the increase of the width-to-narrow ratio, the vorticity distributed in the inclined channel gradually grows. However, this region's vorticity distribution is still less than other regions.

The vorticity is also distributed throughout the whole flow channel for reverse flow. At a low width-to-narrow ratio, the vorticity in the inclined channel is mainly distributed at the entrance, and the vorticity in the middle and latter part of the inclined channel is extremely little. As the width-to-narrow ratio increases, the vorticity in the middle and latter part of the inclined channel increases continuously. It is widely distributed in the case of a high width-to-narrow ratio.

### *Q* criterion

The *Q* criterion is widely used for vortex identification. When *Q* > 0, rotation dominates the flow. The larger the *Q*, the greater the rotation rate of the fluid and the greater the possibility of the existence of vortices. It is based on the decomposition of the velocity gradient tensor ∇*V*, which can be decomposed into a symmetric tensor* A* and an anti-symmetric tensor* B*, representing the deformation and rotation of a point in the flow field. The expressions are

6 $$ \begin{aligned} A & = \frac{1}{2}\left( {\nabla V + \nabla V^{T} } \right) \\ & = \left( {\begin{array}{*{20}c} {\frac{\partial u}{{\partial x}}} & {\frac{1}{2}\left( {\frac{\partial v}{{\partial x}} + \frac{\partial u}{{\partial y}}} \right)} & {\frac{1}{2}\left( {\frac{\partial u}{{\partial z}} + \frac{\partial w}{{\partial x}}} \right)} \\ {\frac{1}{2}\left( {\frac{\partial v}{{\partial x}} + \frac{\partial u}{{\partial y}}} \right)} & {\frac{\partial v}{{\partial y}}} & {\frac{1}{2}\left( {\frac{\partial w}{{\partial y}} + \frac{\partial v}{{\partial z}}} \right)} \\ {\frac{1}{2}\left( {\frac{\partial u}{{\partial {\text{z}}}} + \frac{\partial w}{{\partial x}}} \right)} & {\frac{1}{2}\left( {\frac{\partial w}{{\partial y}} + \frac{\partial v}{{\partial z}}} \right)} & {\frac{\partial w}{{\partial z}}} \\ \end{array} } \right) \\ \end{aligned} $$

7 $$ \begin{aligned} B & = \frac{1}{2}(\nabla V - \nabla V^{T} ) \\ & = \left( {\begin{array}{*{20}c} 0 & {\frac{1}{2}\left( {\frac{\partial u}{{\partial y}} - \frac{\partial v}{{\partial x}}} \right)} & {\frac{1}{2}\left( {\frac{\partial u}{{\partial z}} - \frac{\partial w}{{\partial x}}} \right)} \\ {\frac{1}{2}\left( {\frac{\partial v}{{\partial x}} - \frac{\partial u}{{\partial y}}} \right)} & 0 & {\frac{1}{2}\left( {\frac{\partial v}{{\partial z}} - \frac{\partial w}{{\partial y}}} \right)} \\ {\frac{1}{2}\left( {\frac{\partial w}{{\partial x}} - \frac{\partial u}{{\partial z}}} \right)} & {\frac{1}{2}\left( {\frac{\partial w}{{\partial y}} - \frac{\partial v}{{\partial z}}} \right)} & 0 \\ \end{array} } \right) \\ \end{aligned} $$

The *Q* criterion is based on the second matrix invariant of the velocity gradient tensor, whose expression is

8 $$ Q = \frac{1}{2}\left( {||B||_{F}^{2} - ||A||_{F}^{2} } \right) $$

where || ||_*F*_ denotes the Frobenius parametrization of the matrix^17^.

**Figure 12:**
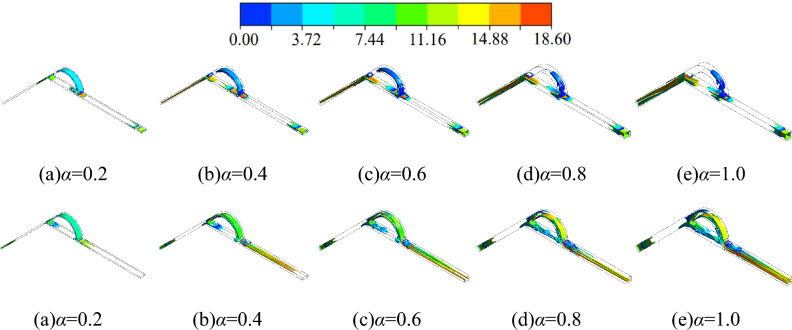
Vortex distribution for different width-to-narrow ratio scenarios (above: forward flow; below: reverse flow).

Based on the *Q* criterion, vortex identification is carried out for flows with different width-to-narrow ratio in this paper, and the incoming velocity is 10 m/s. Setting *Q* = 1.30076 × 10^8^ s^-2^, the vortex distribution is shown in Fig. 12.

The vortex is distributed at the inlet in both forward and reverse flow. In forward flow, the fluid flows from the straight channel, and there are many vortices only at the inlet of the straight channel, with no vortices in any other area of the straight channel. For reverse flow, vortices are also distributed only at the inlet of the inclined channel. In addition, it is found that the change in the width-to-narrow ratio has less effect on the distribution of vortices at the inlet. The vortex at the inlet is widely distributed at any width-to-narrow ratio.

In forward flow, the vortices are distributed at the inlet, the inlet section of the diversion section, the outlet section of the straight diversion section and the entire inclined channel at a high width-to-narrow ratio. As the width-to-narrow ratio decreases, the vortex distributed in the arc diversion section and the inclined channel changes. At *α* = 0.2, the vortex in the arc diversion section increases and is distributed throughout the whole area; the vortex in the inclined channel decreases, and the vortex in the middle and exit sections of the inclined channel disappears. There is no significant difference in the vortex distribution for the other regions inside the valve. In summary, in forward flow, the vortex in the arc diversion section increases as the width-to-narrow ratio decreases, and the vortex in the inclined channel decreases as the width-to-narrow ratio decreases.

It is also found that the vortices in the straight diversion section and the straight channel decrease as the width-to-narrow ratio decreases in reverse flow. For example, at *α* = 1.0, the vortex is distributed throughout the whole straight diversion section and the whole straight channel. While at *α* = 0.2, the vortex is only distributed in the inlet section of the straight diversion section and the inlet section of the straight channel.

In addition, regardless of the forward and reverse flow, the vortex distribution at each width-to-narrow ratio does not differ much in the range of *α* = 0.4 to *α* = 1.0. While in the range of *α* = 0.2 to *α* = 0.4, the difference between the vortex distribution at* α* = 0.2 and that at *α* = 0.4 is obvious. Clearly, the variation of the width-to-narrow ratio in the low width-to-narrow ratio range has a more significant impact on the vortex distribution.

## Conclusion

1. In any case, the straight channel is always the main channel in the forward flow, while the arc channel is not always the main channel in the reverse flow. In the reverse flow, the main channel is still the straight channel in the case of low width-to-narrow ratio with small flow rate.
2. In forward flow, the flow proportion through the straight channel increases with the width-to-narrow ratio increase. In reverse flow, the proportion of flow rate occupied by the arc channel does not increase with the increase in the width-to-narrow ratio. Its maximum proportion of flow rate mainly occurs in the width-to-narrow ratio *α* = 0.4, 0.6 cases.
3. As the width-to-narrow ratio decreases, the pressure drop characteristic in forward and reverse flow is greater, and the pressure change is most pronounced in the case of *α* = 0.2.
4. The difference in pressure variation between forward and reverse flow is most significant in the diversion section. The pressure variation in the forward flow is “∞” shaped in the diversion section, while the trend is “bench” shaped in the reverse flow.
5. Regardless of the forward and reverse flow, the change in flow channel in the low width-to-narrow ratio range (0.2 to 0.4) significantly affects fluid flow, flow rate distribution, pressure drop characteristics, and vortex distribution.

## Data Availability

The data used to support the findings of this study are available from the corresponding author upon request.
